# Unraveling fundamental active units in carbon nitride for photocatalytic oxidation reactions

**DOI:** 10.1038/s41467-020-20521-5

**Published:** 2021-01-12

**Authors:** Chaofeng Huang, Yaping Wen, Jin Ma, Dandan Dong, Yanfei Shen, Songqin Liu, Haibo Ma, Yuanjian Zhang

**Affiliations:** 1 Jiangsu Engineering Laboratory of Smart Carbon-Rich Materials and Device, Jiangsu Province Hi-Tech Key Laboratory for Bio-Medical Research, School of Chemistry and Chemical Engineering, Medical School, Southeast University, Nanjing, 211189 China; 2 Key Laboratory for Green Processing of Chemical Engineering of Xinjiang Bingtuan, School of Chemistry and Chemical Engineering of Shihezi University, Shihezi, 832000 Xinjiang China; 3 School of Chemistry and Chemical Engineering, Nanjing University, Nanjing, 210023 China

**Keywords:** Catalytic mechanisms, Photocatalysis, Photocatalysis

## Abstract

Covalently bonded carbon nitride (CN) has stimulated extensive attention as a metal-free semiconductor. However, because of the complexity of polymeric structures, the acquisition of critical roles of each molecular constituent in CN for photocatalysis remains elusive. Herein, we clarify the fundamental active units of CN in photocatalysis by synthesizing CN with more detailed molecular structures. Enabled by microwave synthesis, the as-prepared CN consists of distinguishable melem (M1) and its incomplete condensed form (M2). We disclose rather than the traditional opinion of being involved in the whole photocatalytic processes, M1 and M2 make primary contributions in light absorption and charge separation, respectively. Meanwhile, oxygen molecules are unusually observed to be activated by participating in the photoexcited processes via electronic coupling mainly to M2. As a result, such CN has a higher activity, which was up to 8 times that of traditional bulk CN for photocatalytic oxidation of tetracycline in water.

## Abstract (web summary)

The acquisition of critical roles of each molecular constituent in carbon nitrides for photocatalysis remains elusive. Here the authors synthesize carbon nitrides with distinguishable units and reveal the roles of the different units in light absorption and charge separation.

## Introduction

Semiconductor photocatalysts have emerged with increasing attention for artificial photosynthesis reactions^1^. Despite enormous advances, developing photocatalysts that have high activity and stability, meanwhile without complex synthetic processes and expensive/toxic elements, is still challenging. Among them, polymeric carbon nitride (CN), consisting of alternatively covalent-bonded C and N atoms, has been stimulated as a prospective candidate with intriguing applications, ranging from solar fuels^2,3^, oxidative pollutant remediation^4,5^, synthetic organic chemistry to very recent optoelectronic biosensing^6–10^, due to its unique electronic structure, exceptional durability under aerobic surroundings and corrosion, and facile preparation using earth-abundant elements^11^. However, owing to slow kinetic processes^12^, a substantial enhancement of CN photocatalytic efficiency is highly envisioned.

Along this line, both the extrinsic approaches (e.g., chemical doping^13,14^, texture engineering^15–18^, and band alignment^19–21^) and the intrinsic routes (e.g., modulation of the condensation degree^22,23^, hydrogen content, crystallinity, and surface defects of CN^24–33^) have been pioneeringly explored. For instance, it was disclosed that low molecular weight CN strengthened the reduction ability^22^, cyanamide defects enhanced co-catalyst interaction/built-in electric field^23^, and N-defects/C–OH terminal groups produced electron trap states^34^. Despite significant advances, the previous studies almost entirely focused on the exploration of a single particular factor/active site influencing the photocatalysis, mainly because of the complexity of polymeric structures. In principle, the acquisition of the critical roles of each featured molecular constituent in CN for the photocatalytic reactions would deepen the understanding of the whole photocatalytic processes and facilitate the precise bottom-up designing and preparing of efficient CN photocatalysts, thus is highly envisioned.

Herein, we clarify the fundamental active units in CN for photocatalytic oxidation reactions through the construction of CN with more defined molecular structures. Enabled by the fast microwave-assisted condensation, the as-prepared CN was verified to mainly consist of melem (M1) and its incompletely condensed form (M2) by a series of careful characterizations. Rather than the traditional opinion of being involved in the whole photocatalytic processes, it was revealed that M1 and M2 were primarily in charge of light absorption and charge separation, respectively; meanwhile, the O_2_ substrate was effectively activated by unusually participating in the photoexcited processes via an electronic coupling. As a result, such a configuration of CN endowed it with an exceptional high activity for photocatalytic oxidation of tetracycline (TC) in water.

## Results

### Preparation of CN_MW-sol_ and CN_MW-ins_

**Fig. 1:**
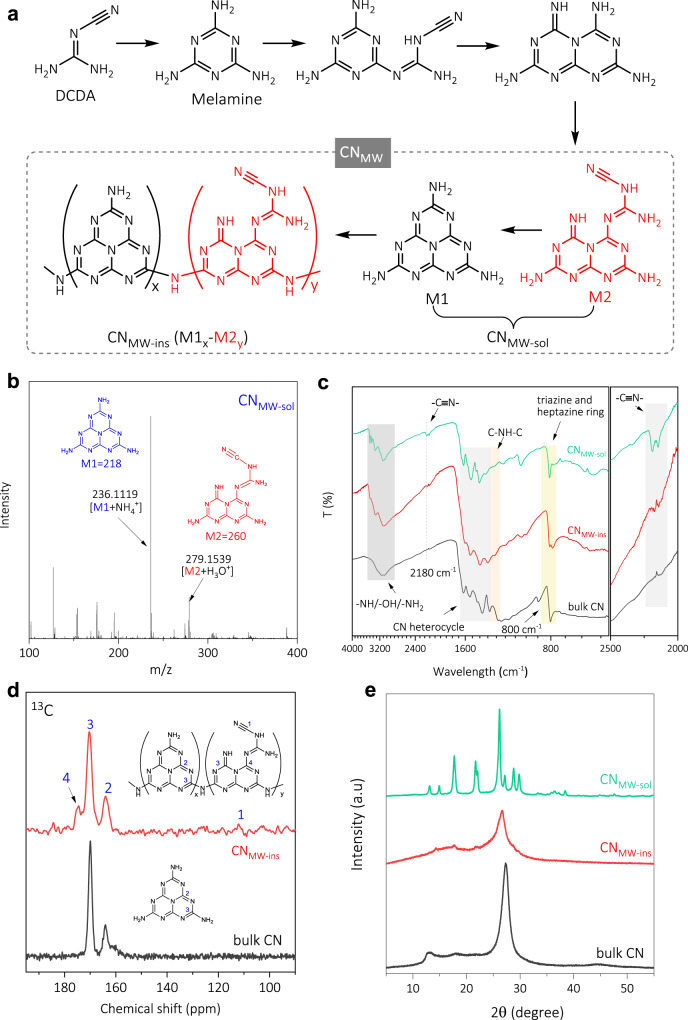
Structural characterizations of CN_MW-ins_ and CN_MW-sol_. **a** Scheme of the general recognized condensation processes for CN_MW_. **b** Q-TOF mass spectrum of CN_MW-sol_. **c** FT-IR of CN_MW-ins_, CN_MW-sol_, and traditional bulk CN. **d** ^13^C NMR spectra of CN_MW-ins_ and bulk CN. **e** Normalized XRD patterns of CN_MW-ins_, CN_MW-sol_, and traditional bulk CN.

A microwave-assisted condensation using ethylene glycol (EG) as the solvent was utilized for synthesizing CN. Compared to the conventional thermal condensation using an electric furnace that generally takes several hours, such fast polymerization in tens of seconds to several minutes which was also reported previously for CN preparation^24,26^. It would promote the synthesis kinetics and maintain the intermediate active sites^35,36^, being favor of disclosure of the fundamental active structure-activity relationship of CN. As a result, a higher yield of pale-yellow powder (CN_MW_) over a yield of 80% was obtained; in contrast, that of traditional bulk CN was only 50–60%. Using ethanol as the solvent, the as-prepared CN_MW_ could be further divided into an insoluble product with a high degree of polymerization (CN_MW-ins_, yield 70.1%) and a soluble product with a low degree of polymerization (CN_MW-sol_, yield 12.4%). The general recognized condensation processes are demonstrated in Fig. 1a^23^, in which the as-prepared CN consisted of melem (M1) and its incompletely condensed form with cyanide termination (M2).

### Structural characterizations of CN_MW-sol_ and CN_MW-ins_

In contrast to bulk CN, CN_MW-sol_ was ready to be disolved, allowing more useful characterization techniques (e.g., the mass spectroscopy) that are rarely used for CN with the aim to disclose more precise molecular structures^37^. According to the general recognized condensation processes^23^, M2 was the last intermediate before forming the M1 framework. To support this speculation, the quadrupole-time of flight (Q-TOF) mass spectrometry (MS), which allows samples to be measured directly, was first used to disclose the possible molecular structure of CN_MW-sol_. As shown in Fig. 1b, the Q-TOF mass spectrum of CN_MW-sol_ illustrated *m/z* [M + NH_4_^+^] of 236.1119 assigning to C_6_N_10_H_6_ (M1, melem, calc.: 218) and *m/z* [M + H_3_O^+^] of 279.1539 attributable to C_7_N_12_H_8_ (M2, incompletely condensed form of M1, calc.: 260). The high-performance liquid chromatography-mass spectrometry profile (HPLC-MS, Supplementary Fig. 1) of CN_MW-sol_ also demonstrated the co-existence of M1 and M2. It should be noted that the intermediates in the early stage of the condensation (theor. molar C/N < 0.56) before the formation of M2 (theor. molar C/N = 0.58) would also be present, but were not likely as the main components in CN_MW-sol_ (molar C/N = 0.58) and CN_MW-ins_ (molar C/N = 0.64), considering results from other characterizations such as the combustion elemental analysis (Supplementary Table 1).

The Fourier transform infrared (FT-IR) spectra of CN_MW-sol_ and CN_MW-ins_ provided further chemical structures information of M1 and M2 (Fig. 1c). It was observed that both of them exhibited typical vibration peaks around 800 and 1200–1700 cm^−1^, corresponding to triazine/heptazine rings and CN heterocycles, respectively, similar to that of bulk CN^9^. Nevertheless, notably, the vibration around 800 cm^−1^ for CN_MW-ins_ exclusively spilt into two comparable peaks, indicating adduct phase of both M1 and M2^38^. Some sharp and broad peaks vibration were also evidently observed for CN_MW-sol_ and CN_MW-ins_ ranging from 2900 to 3600 cm^−1^, which were ascribed to the surface –NH_*x*_ group and physically absorbed H_2_O molecules^25^. More importantly, a typical vibration for cyanide (C ≡ N) stretch was noted at 2180 cm^−1^ for CN_MW-ins_^39^, and it became more apparent for CN_MW-sol_, a reliable indicator for incompletely condensed terminal groups in M2. It should be noted that such a cyanamide-terminated group was also observed in CN by conventional thermal condensation^19,23^, demonstrating the generality of preparing CN by microwave heating with respect to the traditional electric furnace.

The solid-state NMR was also used to understand the nature of the building blocks of CN_MW-ins_ with respect to bulk CN networks. As shown in the ^13^C MAS NMR spectra (Fig. 1d), the presence of heptazine structure in CN_MW-ins_ can be confirmed by the two resolved resonances for heptazine unit at about 164 (2) and 170 (3) ppm, respectively, which is almost identical to that of bulk CN^30^. Moreover, the ^13^C signal for the bay carbon of the incompletely condensed M2 was observed at higher chemical shifts (4). Nevertheless, the ^13^C signal for the carbon atoms in C ≡ N at 112 ppm (see the density functional theory calculation in Supplementary Fig. 2)^19,25,40,41^, generally, was weak, manifesting a low content in CN_MW-ins_, consistent with the result from FT-IR (Fig. 1c) and X-ray photoelectron spectroscopy (XPS, Supplementary Fig. 3) as well^23,30^.

The crystalline texture of CN_MW-ins_ and CN_MW-sol_ was explored by X-ray diffraction (XRD). As a control, the XRD pattern of bulk CN was also measured, which had two peaks at 13.2° and 27.3°, owing to an in-plane structural packing motif and an interlayer stacking reflection, respectively (Fig. 1e)^15^. Similarly, CN_MW-ins_ showed a diffraction peak at ca. 26.6°, but down-shift and widened, indicating a slightly enlarged interlayer spacing; meanwhile, the peak located at 13.2° became weak, suggesting weakened crystallinity^15^. In contrast, CN_MW-sol_ exhibited multiple diffraction peaks, a typical phenomenon for small organic molecules (Supplementary Fig. 4). Consistent with the solubility, it depicted that the condensation degree and the molecular size of CN_MW-sol_ were smaller than CN_MW-ins_. In a sense, CN_MW-sol_ can be regarded as an intermediate of CN_MW-ins_ during the condensation. To further confirm this assumption, the C/N molar ratio of CN_MW-sol_ was examined to be 0.58 by combustion elemental analysis (Supplementary Table 1), which was smaller than that of CN_MW-ins_ (0.64). Moreover, these two ratios located between that of melamine (0.51) and bulk CN (0.68), indicating a moderate depletion of nitrogen during the condensation processes^42^.

Therefore, all these structural explorations supported the recognized condensation processes for CN_MW_ (Fig. 1a) with the proposed molecular constituents of M1 and its incompletely condensed form of M2. It would be discussed in the following text that these two primary units, i.e., M1 and M2, played respective roles in light absorption and charge separation/migration in the showcase photocatalytic TC oxidation reaction.

### Photocatalytic TC oxidation activity

Apart from widely explored solar fuels generation, the photogenerated electron and hole are valuable in efficient water purification, which is one of the primary considerations for health and safety^43^. For instance, TC is generally recognized as a typical contamination of pharmaceuticals and personal care products (PPCPs) in the environment, and induces the development of antibiotic-resistant pathogens and cause serious problems for human health^44^. Therefore, seeking an economical and environmental-friendly method to remove the PPCPs is essential. Notably, the photocatalytic molecular oxygen activation occurs in a timescale approximately six orders of magnitude faster than molecular oxygen evolution, acknowledged in both natural and artificial photocatalysts^25,27–30^. In this context, the attempt to remove PPCPs by CN photocatalyst with the participation of molecular oxygen was intriguing.

**Fig. 2:**
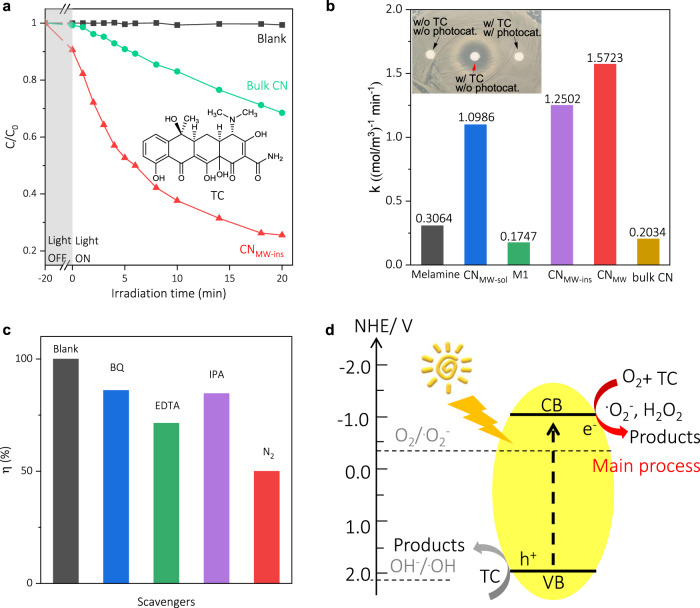
Photocatalytic sanitation of TC-contaminated water by CN. **a** Absorbance of TC at 357 nm as a function of time during photocatalytic oxidation reaction (>400 nm) using bulk CN and CN_MW-ins_ catalyst. **b** Pseudo-second-order reaction kinetics for TC sanitation using different photocatalysts. Inset: bactericidal halo test. “w/” and “w/o” denote “with” and “without,” respectively. **c** Photocatalytic activity of CN_MW-ins_ for TC sanitation in the presence of different scavengers. **d** Proposed mechanism of photocatalytic oxidation of TC.

The photocatalytic oxidation of TC by different CN was investigated. As shown in Fig. 2a, the absorbance of TC was reduced by 10% in the presence of CN_MW-ins_ under the absorption–desorption equilibrium in the dark, which was larger than that by bulk CN, due to the stronger electrostatic interaction between of TC and CN_MW-ins_ (zeta potential of 37 mV, Supplementary Fig. 5). Moreover, under identical conditions, the CN_MW-ins_ had a more notable photocatalytic oxidation ability than bulk CN. For instance, after irradiation for 20 min, only ca. 25% of TC was unreacted by CN_MW-ins_, while that was ca. 70% by bulk CN. As a control, the TC concentration remained almost unchanged without any photocatalysts, which demonstrated that TC was stable under irradiation, and the oxidation activity was exclusively derived from the photocatalysts. For a quantitative comparison of kinetics, the pseudo-second-order rate constant (*k*) was calculated. Figure 2b showed the *k* for CN_MW-ins_ (1.2502 mol^−1^ m^3^ min^−1^) was ca. 6 times of that for bulk CN (0.2034 mol^−1^ m^3^ min^−1^). CN_MW-sol_ also demonstrated an apparent photocatalytic activity (*k* = 1.0986 mol^−1^ m^3^ min^−1^), which was further boosted after cooperation with CN_MW-ins_ as CN_MW_ (*k* = 1.5723 mol^−1^ m^3^ min^−1^, Supplementary Fig. 6). The even higher activity of the CN_MW_ sample may be explained by the isotype heterojunction of matched electronic band structures between CN_MW-ins_ and CN_MW-sol_ that promoted the charge separation (Supplementary Fig. 7)^45–47^, rather than the varieties in the geometric structures (see SEM/TEM images in Supplementary Fig. 8). In contrast, only a marginal photocatalytic activity was observed for melamine, M1, and bulk CN, demonstrating the importance of coupling M1 and M2 in synergistically boosting the photocatalytic activity.

The bactericidal halo test was further conducted on Luria–Bertani agar to investigate the antibacterial activity of the oxidized TC by CN_MW_. As depicted in Fig. 2b inset, the treated TC solution exerted no inhibitory effect on Staphylococcus aureus, as evidenced by the almost invisible inhibition zones. It indicated that the photocatalytic oxidation by CN_MW_ would effectively covert TC into environmental and bio-safe molecules. Besides, CN_MW_ maintained a good photocatalytic performance during three reaction cycles, indicative of reasonable stability (Supplementary Fig. 9). It should be noted that the similar activity trends among different CN samples such as CN_MW_ and bulk CN were also observed for the photocatalytic oxidation of other substrates, such as Azure B (Supplementary Fig. 10)^9^.

### Mechanism study of CN_MW_ in photocatalytic oxidation

To understand the photocatalytic mechanism, the trapping experiments were carried out to explore the possible intermediate reactive species. It was observed that by adding superoxide, hole, ·OH, or O_2_ scavengers, the oxidation efficiency of CN_MW-ins_ demonstrated a various degree of inhibition (Fig. 2c)^48^. Remarkedly, among them, the oxidation activity was drastically restrained when N_2_ was purged into the reaction solution, revealing that O_2_ was an important co-substrate and the activation of O_2_ by reduction via photogenerated electrons into O_2_·^–^ and H_2_O_2_ (Supplementary Fig. 11) was the major step in the oxidation of TC (Fig. 2d)^49,50^.

The UV–Vis spectra (Supplementary Fig. 7a) and photoluminescent spectra (Supplementary Fig. 12) of CN_MW-ins_ and CN_MW-sol_ were also measured. It was observed that CN_MW-ins_ had more light absorption than CN_MW-sol_. Considering CN_MW-ins_ had a higher ratio of M1 than CN_MW-sol_, it suggested that M1 had a more substantial light harvesting with respect to M2. Moreover, it was noted that even CN_MW-ins_ also demonstrated a much less light absorption than bulk CN, verifying the better activity of CN_MW-ins_ with respect to bulk CN was not ascribed to the absorption of more photons. As CN_MW-ins_ contained more M2 than bulk CN, it indicated that M2 may play an essential role in accelerating the charge separation. In this sense, the hole- and electron-extraction properties were evaluated by measuring anodic and cathodic photocurrents, respectively, in the presence of an electron donor (triethanolamine, TEOA), assuming the maximum photocurrent can be obtained without any hole- or electron-transfer limitations^51,52^. As shown in Supplementary Fig. 13, both anodic and cathodic photocurrents at CN_MW-ins_ were less improved with respect to bulk CN by adding TEOA, indicating superior hole- and electron-extraction properties contributed by M2 during the reaction in aqueous solution.

**Fig. 3:**
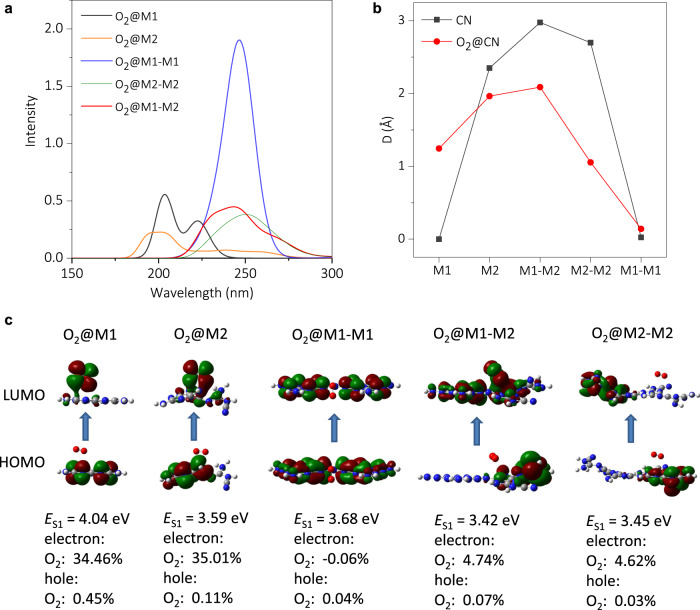
Computational analysis of M1, M2, and electron coupling. **a** Simulated absorption spectra of O_2_@CN. **b** Centroids distance (*D*) of electrons and holes in the lowest singlet electronic excited state (S_1_) of CN and O_2_@CN. **c** Isosurface plots of the highest occupied molecular orbital (HOMO) and the lowest unoccupied molecular orbital (LUMO) of different O_2_@CN. The excitation energy (*E*_S1_) and the percentage of O_2_ contribution to electrons and holes in S_1_ are shown in the bottom panel.

For more comprehensive mechanism insights of the increased photocatalytic activity, particularly with the critical role of M1/M2 and the participation of oxygen substrates, quantum chemical calculations of monomers, such as M1/M2, M1–M1, M2–M2, and the M1–M2 bonding patterns, were performed (Supplementary Fig. 14). Due to the intrinsic higher conjugation, the simulated absorption spectra showed that M1 had a higher intensity than M2, no matter with (Fig. 3a) or without (Supplementary Fig. 15) interaction with O_2_ substrate. It became more evident when two M1 forms a covalent-bonded dimer; after coupling to M2, a suppressed intensity of absorption was observed for M1–M2, but still stronger than M2 monomer or dimer. Consistently, presumably owing to a higher ratio of M1, more light absorption of CN_MW-ins_ compared to that of CN_MW-sol_ was also observed in experiments (Supplementary Fig. 7a). These facts exclusively demonstrated the primary role of M1 in light harvesting. Besides, it was also noted that owing to the extension of conjugation, the M1–M2 dimer had an extended absorption edge in longer wavelength with respect to M1 monomer or dimer, indicative of an easier excitation.

In addition to light harvesting, the charge separation/migration was also essential. For this, the spatial distribution of electron and hole populations of M1, M2, and their dimers in the lowest singlet electronic excited state (S_1_) without and with the participation of O_2_ were simulated (Supplementary Fig. 16) and quantified (Supplementary Table 2). Interestingly, a strong electronic coupling interaction by O_2_ substrate was observed. For instance, the electrons and holes of M1 alone were distributed on the whole framework with a large degree of overlapping, while with the participation of O_2_ the overlap was noticeably suppressed, making the separation became more effective (see the quantitative centroids distance in Fig. 3b). Note that the substrates-associated electronic coupling to the light absorption and charge separation/migration was essential, especially in photocatalytic oxidation reactions, but has been rarely studied and poorly understood. Moreover, due to the strong electron deficiency of cyano group in M2, the electron–hole separation became evident in M2 and more significant in M1–M2.

The electron distribution of the frontier molecular orbitals of all O_2_@CN in S_1_ was further depicted in Fig. 3c. It was observed that the excitation energy of S_1_ (*E*_S1_) in M1–M2 was the lowest, indicating the easiest excitation. Notably, for the highest occupied molecular orbital, the electrons were only localized on the CN molecules. In contrast, for the lowest unoccupied molecular orbital (LUMO), the electrons in the O_2_@M1 and O_2_@M2 were mainly contributed by O_2_, and that in O_2_@M1–M1 and O_2_@M2–M2 systems were primarily distributed in CN framework.

**Fig. 4:**
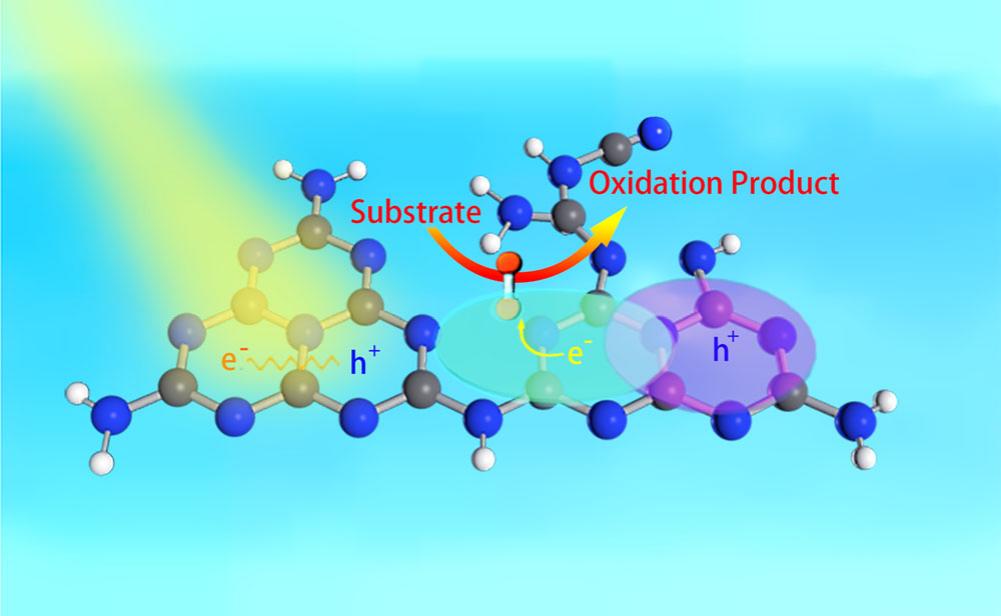
Roles of each molecular constituent in carbon nitrides. Scheme of the light harvesting and the spatial distribution of electron/hole on M1–M2 framework with the electron coupling of O_2_.

Interestingly, the electrons in O_2_@M1–M2 were distributed in both O_2_ and CN framework (primarily of M2). Moreover, the quantified contribution of O_2_ in M1–M2 (4.74%) was greater than that in O_2_@M1–M1 (−0.06%). Thus, compared to M1–M1, M1–M2 was more likely to be excited along with a higher contribution of O_2_ in LUMO, i.e., more conducive to activate O_2_ in the following oxidation reaction. Such trends could also be observed by the uneven distribution of the electron density of the whole structure with M1 and M2 (Supplementary Fig. 17). Notably, the more detailed structures and properties of the CN photocatalysts and the oxidation products would be very interesting and deserve a future investigation. Briefly, the photoexciting processes for O_2_@M1–M2 were summarized in Fig. 4. It is highly envisioned that the acquisition of the critical roles of each featured molecular constituent in CN for the photocatalytic reactions would reshape the understanding of the whole photocatalytic processes and facilitate the precise bottom-up designing and preparing of efficient CN photocatalysts.

## Discussion

In summary, we report the preparation of CN_MW_ consisted of molecular constituents of melem (M1) and its incomplete condensed form with cyanide termination (M2) by a microwave-accelerated condensation in tens of seconds. Both experiments and theoretical analysis revealed that M1 and M2 intriguingly made a predominated contribution in light absorption and charge separation, respectively. Meanwhile, the oxygen substrate was surprisingly observed to participate in the photoexcited processes via an electronic coupling process, mainly to M2 in the final CN_MW_. As a result, an exceptional boosted photocatalytic activity for TC oxidation by CN_MW_ was observed up to eight times over the conventional bulk CN. The more detailed understanding of the critical roles of each fundamental active unit and their synergistic effects would substantially pave the way for rational bottom-up preparation and practical applications of CN and other emerging polymeric photocatalysts as well in energy, environmental remediation, and beyond, such as prospective optoelectronic biosensing.

## Methods

### Reagent

The following chemicals were obtained as indicated: dicyandiamide (DCDA, 99%, Sigma-Aldrich), TC (98%, Aladdin), isopropanol (IPA, 99.8%, Aladdin), ethylenediaminetetraacetic acid disodium salt (EDTA, 99%, Sinopharm chemical Reagent), benzoquinone (BQ, 99%, J&K), and EG (99%, Sinopharm chemical Reagent). All chemicals were used without further purification, unless otherwise specified. Ultrapure water (18.2 MΩ cm) was obtained from a Smart2 water purification system (Thermo Scientific, USA).

### Preparation of bulk CN and M1

Bulk CN was prepared by heating DCDA for 4 h to 550 °C and kept at this temperature for 4 h in air. The final product was ground into fine powder and used without further purification. The M1 sample was prepared, according to the previous study of polymerization kinetics of bulk CN. Briefly, it was synthesized by condensation of DCDA at 390 °C in a muffle furnace.

### Microwave-assisted synthesis of CN_MW_

First, 20 g of DCDA precursor was added into 200 mL of EG (owning a high loss factor tan *δ* of 1.350, due to efficient electromagnetic radiation absorption and rapid heating, boiling point: 198 °C) under stirring at 70 °C for 15 min, at the end of which, the Tyndall effect was normally not observed, indicating the formation of a true solution instead of a colloidal system. Second, 5 mL of DCDA/EG solution was added into a crucible and placed into a microwave reactor (700 W, M1-L213B, 2.45 GHz, Midea, China), and then irradiated for several 10 s (typically of 90 s). The resulting sample was denoted as CN_MW_. Then, CN_MW_ was stirred in ethyl alcohol for several hours to separate the insoluble part denoted as CN_MW-ins_ and soluble part denoted as CN_MW-sol_.

### Photocatalytic oxidation of tetracycline

Briefly, 100 mg of photocatalyst was added in a quartz tube (5 × 5 × 5 cm, 20 mL) of 0.05 mg/mL TC aqueous solution. First, the suspension was stirred for 20 min in dark to ensure the establishment of adsorption equilibrium. Afterwards, the quartz tube was top-irradiated under full light by using a 300 W Xe lamp (CEL-HXUV300E, China) with a short-pass filter cutting off lights of wavelength less than 400 nm. 0.4 mL of suspension was extracted and centrifuged at certain time intervals. The photocatalytic oxidation efficiency of TC at 357 nm was analyzed on the UV–Vis spectrophotometer. In order to investigate the main oxidation species of the photocatalytic reaction, IPA, EDTA disodium salt, and BQ were selected as the scavengers for ·OH, h^+^, and ·O^2−^, respectively. The concentration of these scavengers was 0.1 mM.

### Characterization

The FT-IR spectra were recorded with a Nicolet 4700 (Thermo, USA) equipped with an attenuated total reflection setup. The UV–Vis absorption spectra were obtained with Cary 100 (Agilent, Singapore) and BaSO_4_ as a reference. The XRD measurements were recorded on SmartLab diffractometer (Rigaku, Japan). The photoluminescence spectra were performed (Fluoromax-4, Horiba Jobin Yvon, Japan). Elemental analysis was performed on a Vario EL elemental analyzer (Germany). The zeta potential analyzer was obtained (Brookhaven Instruments Corporation, USA). XPS analysis was obtained by using a Thermo ESCALAB 250Xi instrument with a monochromatized Al_Kα_ X-ray source (hν = 1486.6 eV). MS was operated in electrospray ionization (ESI) positive mode of accurate liquid chromatography/MS Q-TOF (LCMS-9030, Shimadzu, Japan). HPLC-MS was performed in ESI positive mode (U3000/LCQ Fleet, Thermo Scientific, USA). Flow rate was set at 0.6 mL/min: 10% of phase A (H_2_O) and 90% phase B (MeOH), with column oven maintained at 35 °C. ^13^C NMR spectra were carried on a JNM-ECZ600R spectrometer (JEOL, Japan) and all spectra were referenced to adamantane.

### Computational details

The ground state geometries of all CN molecules and O_2_@CN systems were optimized by DFT at the M06-2X/6-31G(d,p) level. The vibrational frequencies were calculated at the same level to confirm the optimized configurations were the minimum energy points. Based on the optimized geometries, the absorption spectra were simulated by TDDFT with M06-2X functional and 6-31G(d,p) basic set. All the above calculations were implemented in the Gaussian 16 program^53^. Besides, to obtain an insight into the characters in the excited state of electrons, the electron–hole distribution of the lowest singlet electronic excited state (S_1_) in all configurations was analyzed in detail by using Multiwfn program^54^.

## Supplementary information

Supplementary Information

## Data Availability

The data supporting the findings of this study are available within the article and its Supplementary Information, as well as from the corresponding author on reasonable request.
