# Supplementary material for: Unraveling fundamental active units in carbon nitride for photocatalytic oxidation reactions
Source: Nat Commun. 2021 Jan 12;12:320. doi: 10.1038/s41467-020-20521-5 (PMC7804405; doi:10.1038/s41467-020-20521-5)
Supplement: Supplementary file 1 — Supplementary Information [file 41467_2020_20521_MOESM1_ESM.pdf]

Supplementary Information

Unraveling Fundamental Active Units in Carbon Nitride for Photocatalytic Oxidation Reactions

Chaofeng Huang,[‡a, b] Yaping Wen,[‡c] Jin Ma[‡a], Dandan Dong,[b] Yanfei Shen,[a] Songqin Liu,[a] Haibo Ma*[c] and Yuanjian Zhang *[a]

[a] Jiangsu Engineering Laboratory of Smart Carbon-Rich Materials and Device, Jiangsu Province Hi-Tech Key Laboratory for Bio-Medical Research, School of Chemistry and Chemical Engineering, Medical School, Southeast University, Nanjing 211189, China. E-mail: Yuanjian.Zhang@seu.edu.cn

[b] School of Chemistry and Chemical Engineering of Shihezi University, and Key Laboratory for Green Processing of Chemical Engineering of Xinjiang Bingtuan, Shihezi, Xinjiang 832000, China.

[c] School of Chemistry and Chemical Engineering, Nanjing University, Nanjing 210023, China. E-mail: haibo@nju.edu.cn

‡ These authors contributed equally.

Table of Content

Supplementary Methods

Photoelectrochemical (PEC) experiments

Photocatalytic oxidation of Azure B

Recyclability of CN_{MW}

Supplementary Characterization Figures

Supplementary Figure 1 HPLC-MS spectra

Supplementary Figure 2 Simulated ^{13}C NMR spectrum of M2 by DFT calculation

Supplementary Figure 3 High-resolution C1s and (b) N1s XPS spectra

Supplementary Figure 4 XRD patterns

Supplementary Figure 5 Zeta potentials

Supplementary Figure 6 Plot of $1/C-1/C_0$ as a function of irradiation time for photocatalytic oxidation of TC

Supplementary Figure 7 UV-Vis spectra, XPS VB scan spectra and calculated energy diagram

Supplementary Figure 8 SEM/TEM images

Supplementary Figure 9 Cyclic catalytic efficiency and XRD patterns

Supplementary Figure 10 Photocatalytic oxidation of Azure B

Supplementary Figure 11 UV-vis absorption of TMB aqueous solution with and without CN_{MW} photocatalyst with irradiation

Supplementary Figure 12 PL spectra

Supplementary Figure 13 Current–potential curve under chopped light

Supplementary Mechanism study of CN in photocatalytic oxidation

Supplementary Figure 14 Optimized geometries of CN molecules

Supplementary Figure 15 Absorption spectra of CN molecules

Supplementary Figure 16 Smooth description of spatial distribution of electron and hole population

Supplementary Figure 17 Electrostatic surface potentials mapped onto a surface of total electron density for all the investigated molecules.

Supplementary Tables

Supplementary Table 1 Combustion elemental analysis

Supplementary Table 2 Centroid distance of electron-hole (*D*), overlap integral (S_r), index of separation degree (t) and coulomb attraction energy (E_c) of CN and O_2@CN molecules.

Supplementary References

Supplementary Methods

Photoelectrochemical (PEC) experiments

The photoelectrodes were prepared by spreading aqueous slurries of various CN-based materials over 0.25 cm^2 of ITO glass substrate using adhesive tapes as spaces. The suspension was preparation by grinding 8 mg of CN-based materials with 60 μL of 0.1wt.% Nafion. Then the photoelectrodes were annealed in air at 120 °C and kept at this temperature for half an hour. The photoelectrochemical measurements were performed in a conventional three-electrode system in a quartz tank with a platinum wire as the counter electrode and Ag/AgCl (saturated KCl) electrode as the reference electrode. Illumination through the ITO back-side illumination was used. The power density of the incident light was calibrated to 1000 W/m^2 at the surface of CN-based photoelectrodes by a 150 W Xe lamp (Beijing NBeT Co., Ltd.) with optical filter (>400 nm). All illuminated areas were 0.25 cm^2. Photocurrent measurements were performed in a 0.1 M KCl solution at a scan rate of 10 mV/s. The current difference between the light and the dark was defined as the net photocurrent.[1]

Photocatalytic oxidation of Azure B

Briefly, 100 mg of photocatalyst was added in a quartz tube (5×5×5cm, 20 mL) of 0.05 mg/mL Azure B aqueous solution (AB). Firstly, the suspension was stirred for 20 min in dark to ensure the establishment of adsorption equilibrium. Afterwards, the quartz tube was top-irradiated under full light by using a 300 W Xe lamp (CEL-HXUV300E, China) with a short-pass filter cutting off lights of wavelength less than 400 nm. 0.4 mL of suspension was extracted and centrifuged at certain time intervals. The photocatalytic oxidation efficiency of AB at 604 nm was analyzed on the UV-vis spectrophotometer.

Recyclability of CN_{MW}

After each photocatalytic oxidation of TC, the equal amount of TC was added for next recycling test.

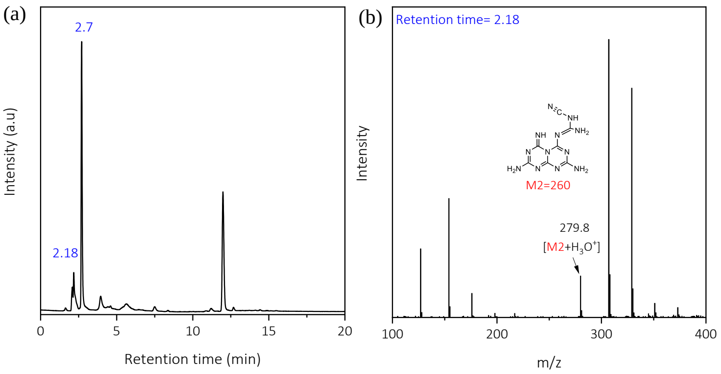

Supplementary Figure 1 HPLC-MS spectra of $CN_{MW\text{-}sol}$.

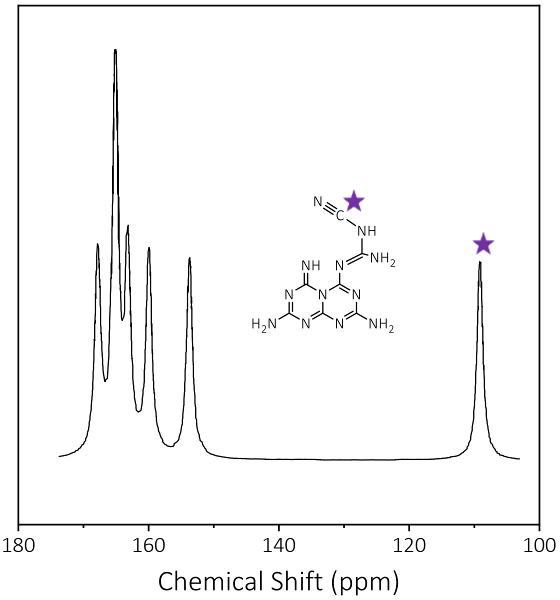

Supplementary Figure 2 Simulated ^{13}C NMR spectrum of M2 by DFT calculation.

Density functional theory (DFT) calculations were performed by using Gaussian 16 software package (revision A.03). Before NMR calculation, the structural models were optimized at B97-2/6-311g (d, p) level with frequency examination. And the ^{13}C NMR spectrum of repetitive units, M1 and M2, were calculated at the B97-2/def2-QZVP level.[2]

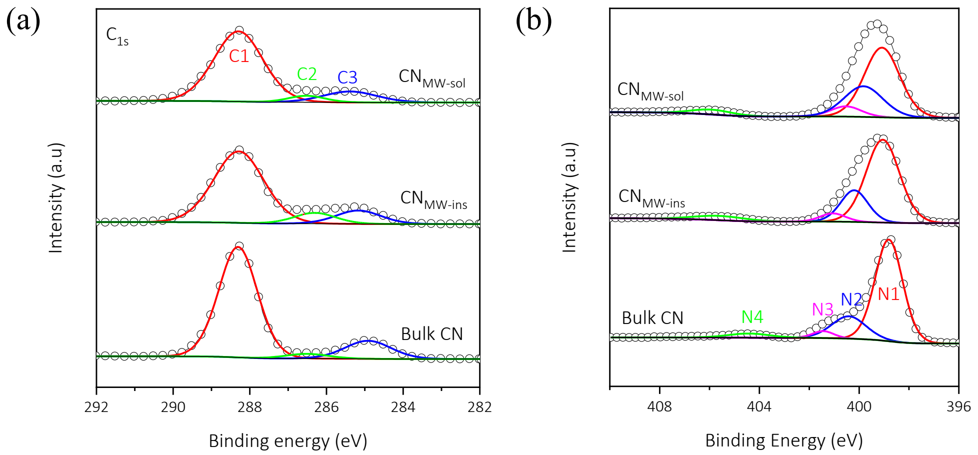

Supplementary Figure 3 (a) High-resolution C1s and (b) N1s XPS spectra of bulk CN, $CN_{MW\text{-}ins}$ and $CN_{MW\text{-}sol}$.

X-ray photoelectron spectroscopy (XPS) was recorded to identify the different binding characteristics of nitrogen and carbon atoms in $CN_{MW\text{-}ins}$, $CN_{MW\text{-}sol}$, and bulk CN. The high-resolution N1s spectra could be deconvoluted into the sp^2 C=N bond (398.8 eV, tri-s-triazine rings), N-$(C)_3$ (400.4 eV, bridging N atoms), C-N-H (401.4 eV, bonded with H atoms) and charge effect (404.4 eV).[3] Meanwhile, high-resolution C1s spectra showed three main peaks assigned to random adventitious carbon contamination (284.6 eV), C-NH_x bond (286.4 eV)[4] and aromatic -C=N (288.3 eV, tri-s-triazine rings), respectively. It can be seen that CN_{MW} had a lower polymerization with regard to the conventional bulk CN.

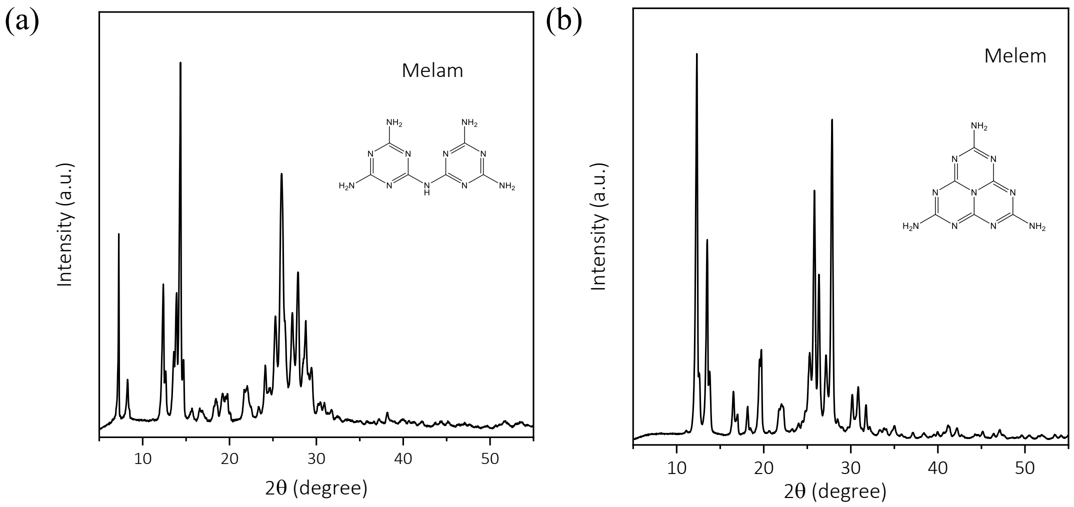

Supplementary Figure 4 XRD patterns of (a) melam and (b) melem (M1).

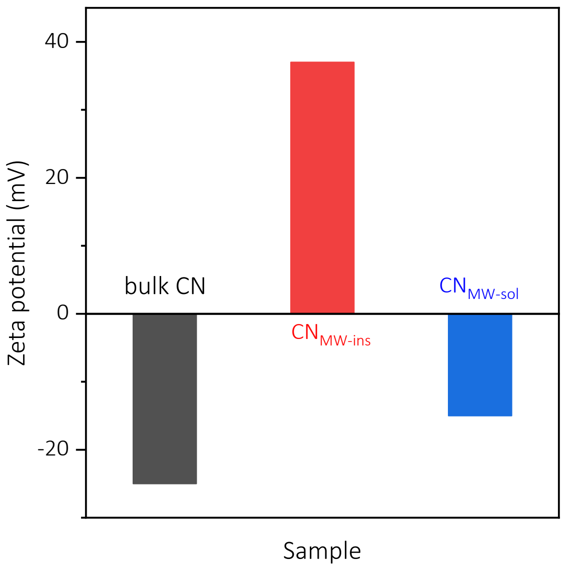

Supplementary Figure 5 Zeta potentials of bulk CN, $CN_{MW\text{-}ins}$ and $CN_{MW\text{-}sol}$ dispersion in aqueous solution.

The positive zeta potential of $CN_{MW\text{-}ins}$ could be explained by the protonation of the -NH_x groups that anchored to the alkyl chain in M2. In contrast, the terminal -NH_x groups on bulk CN has poor protonation ability, due to the delocalization of the unpaired electron in N to the conjugated tri-s-triazine framework. Interestingly, for bulk CN, a negative zeta potential for bulk CN is often observed.[5, 6] The exact mechanism has not been very clear yet, and is generally supposed to the absorption of hydroxyl ions in aqueous solution. For $CN_{MW\text{-}sol}$, the M2 portion would partially dissolve in the solution during the sample preparation via ultrasonication for the zeta potential measurements, making it less competitive to the absorption of hydroxyl ions that are negatively charged.

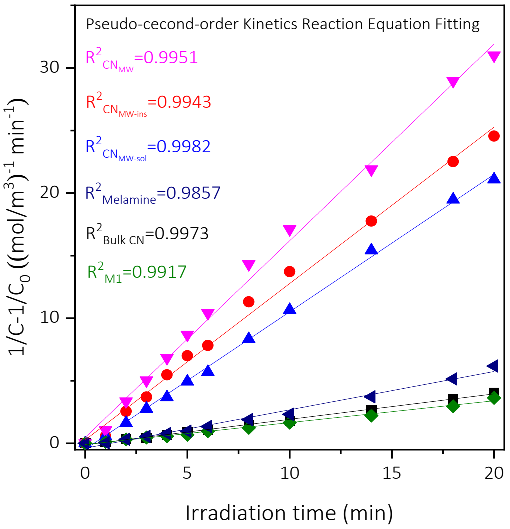

Supplementary Figure 6 Plot of $1/C-1/C_0$ as a function of irradiation time for photocatalytic oxidation of TC using CN_{MW}, bulk CN and other control photocatalysts.

A higher photocatalytic oxidation of TC by melamine than bulk CN was observed, in which the homogeneous dissolution of melamine in the aqueous solution was supposed to be a favorable factor.[7] Nonetheless, it should be noted that the activity of melamine was still rather poor with respect to CN_{MW}, owing to marginable absorption of visible light.

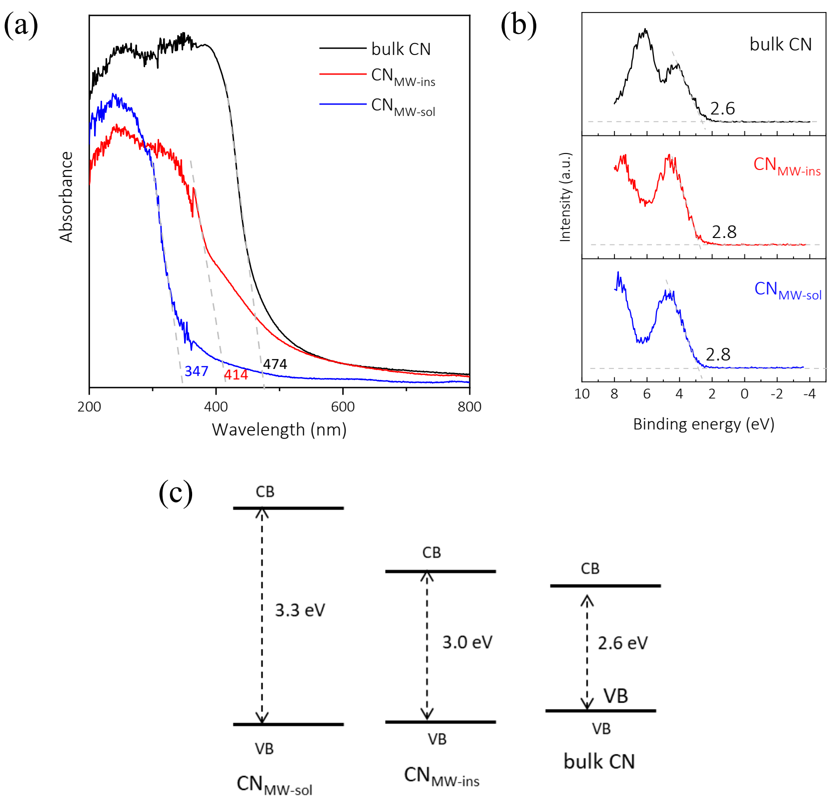

Supplementary Figure 7 (a) UV-Vis spectra, (b) XPS VB scan spectra and (c) calculated energy diagram of the bulk CN, CN$_{MW-ins}$ and CN$_{MW-sol}$ samples.

As disclosed by the trapping techniques, the photooxidation of TC was predominately driven by the activation of O_2 into $O_2^{\bullet -}$ and H_2O_2 via the reduction using the photogenerated electrons. In this sense, the higher CB position would be indicative of stronger oxidation ability. As calculated by using UV-Vis spectra and XPS VB scan spectra, the order of the oxidation ability followed as: CN$_{MW-sol}$ > CN$_{MW-ins}$ > bulk CN. Nonetheless, it should be aware that except for the oxidation ability, the overall photocatalytic oxidation activity is also influenced by the light absorption, electron donating ability of substrates, dispersibility and other factors as well.

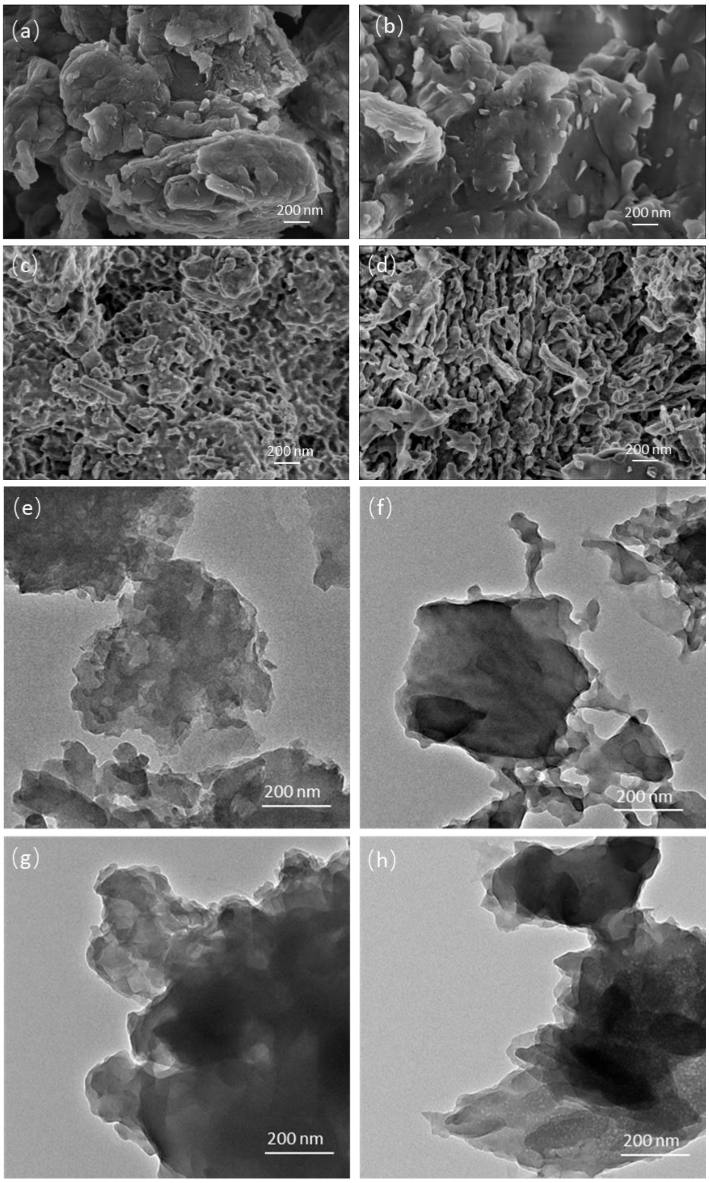

Supplementary Figure 8 SEM images of bulk CN (a), CN_{MW} (b), $CN_{MW\text{-}ins}$ (c) and $CN_{MW\text{-}sol}$ (d); TEM images of bulk CN (e), CN_{MW} (f), $CN_{MW\text{-}ins}$ (g) and $CN_{MW\text{-}sol}$ (h).

From the SEM and TEM images, it can be seen that the stacked structure of bulk CN was retained in the as-prepared CN_{MW}. Nonetheless, many macro-holes were found in the $CN_{MW\text{-}ins}$, which was supposed to be generated by extracting the solvable $CN_{MW\text{-}sol}$ using the solvent. From another point of view, it also indicated that $CN_{MW\text{-}ins}$ and $CN_{MW\text{-}sol}$ were seamlessly coupled. The surface area of CN_{MW} (6.3 m^2/g), and $CN_{MW\text{-}ins}$ (37.6 m^2/g) were also measured by N_2 absorption/desorption and compared with that of bulk CN (13.3 m^2/g). It was found that the surface areas of them were far less proportional to the photocatalytic activity, indicating the surface area did not played a key role in this work. Besides, it should be mentioned that the morphology of the solid $CN_{MW\text{-}sol}$ largely depended on the precipitation method from the solution.

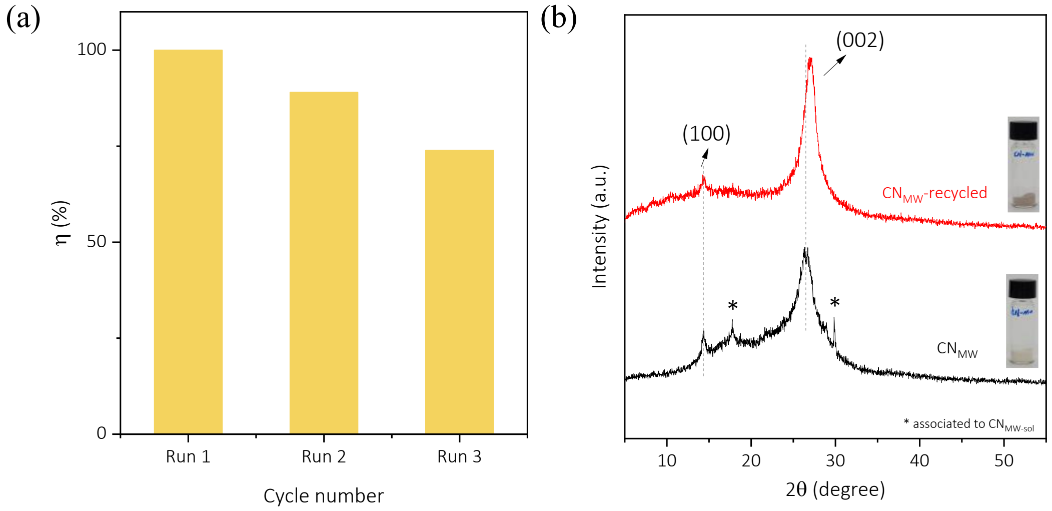

Supplementary Figure 9 (a) Cyclic catalytic efficiency of the CN_{MW} catalyst in the photocatalytic oxidation of TC. (b) XRD patterns of CN_{MW} before and after the photocatalytic oxidation of TC. Inset: Photos of CN_{MW} before and after the photocatalytic oxidation of TC.

Some decrease in photocatalytic activity was observed in Fig. S9a, but still fair, as remaining 74% after three cycles. It was presumably ascribed to the partial absorption of TC oxidation product on the surface of CN_{MW} (the color of the catalyst gradually became greyish, see the photo in Figure S9b inset), rather than the photocatalytic corrosion of CN_{MW} itself. To verify this assumption, we examined the XRD of the CN_{MW} sample after the recycle. It was found that except for the disappearance of some minor peaks (*) and slight up-shift of (002) diffraction peak, the most representative (100) and (002) diffraction peaks of CN_{MW} were mostly retained (Supplementary Figure 9b).[8] Note that the XRD pattern of $CN_{MW\text{-}sol}$ exhibited exactly such two minor diffraction peaks and a (002) diffraction peak at smaller angle (Supplementary Figure 9b), the above change in XRD results can be explained by the dissolution of $CN_{MW\text{-}sol}$ from CN_{MW} during the reaction in aqueous solution and recycling processes, which weakened the contribution of the diffraction from $CN_{MW\text{-}sol}$ in CN_{MW}. Indeed, due to the graphitic structures and very low valance band position (close to the standard potential of Au^{3+}/Au),[3] carbon nitrides are "noble", that is, cannot be easily oxidized (no noticeable oxygen functionalities were observed on the surface, unless additional chemicals were adopted),[9] thus having high chemical and thermal stability (up to 500 °C in air) and being widely explored in photocatalytic reactions. Thus, the stability of CN_{MW} was still rather good against photocatalytic corrosion of itself.

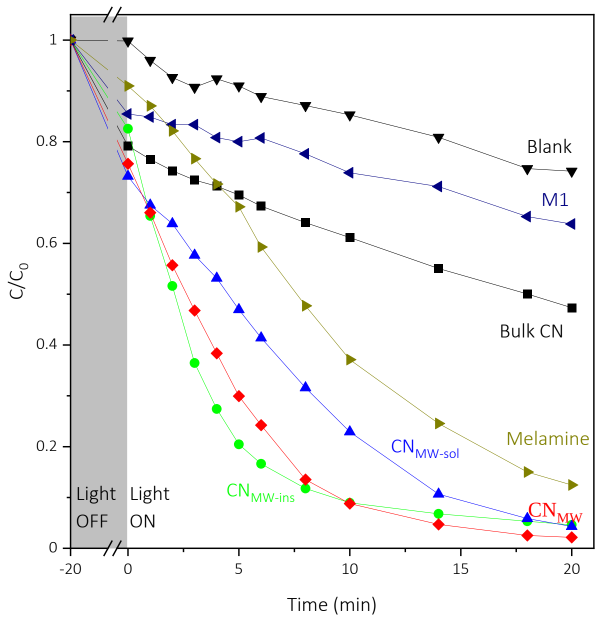

Supplementary Figure 10 Absorbance of Azure B at 604 nm as a function of time during photocatalytic oxidation reaction (>400 nm) using CN_{MW}, bulk CN and other control photocatalysts.

The similar activity trends among different carbon nitride samples such as CN_{MW} and bulk CN was also observed for the photocatalytic oxidation of other substrates, such as Azure B. Nonetheless, the photocatalytic oxidation activities also correlated to the electron donating ability of substrates and their surface properties, which appeals for more future investigations.

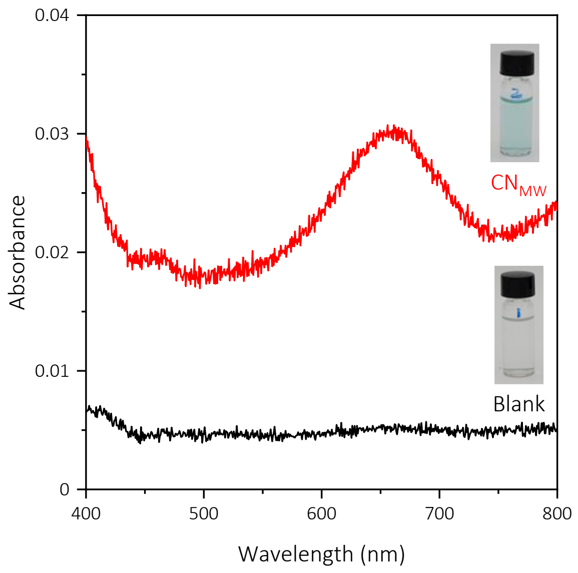

Supplementary Figure 11 UV-Vis absorption of TMB aqueous solution with (red) and without (black) CN_{MW} photocatalyst with irradiation.

To check whether H_2O_2 was produced or not, the CN_{MW} dispersion (5 mg/mL) was irradiated for 40 min. After that, it was kept in dark for 30 min to eliminate the interference of reactive oxygen species with short lifetime. Horseradish peroxidase (HRP) and 3,3′,5,5′-Tetramethylbenzidine (TMB) was then added and incubated for 5 min after the removal of the catalyst by centrifugation. It was supposed that if H_2O_2 was present, colorless TMB would be catalytically oxidized into blue TMB_{ox} by HRP.[10] As shown in the UV-Vis spectra below, the typical absorbance of TMB_{ox} in case of CN_{MW} was improved with respect to the control pure water, which could also be indicated by a visual color change from colorless to blue of the solution (see the inset photo). Thus, H_2O_2 was produced during the photocatalytic activation of O_2.

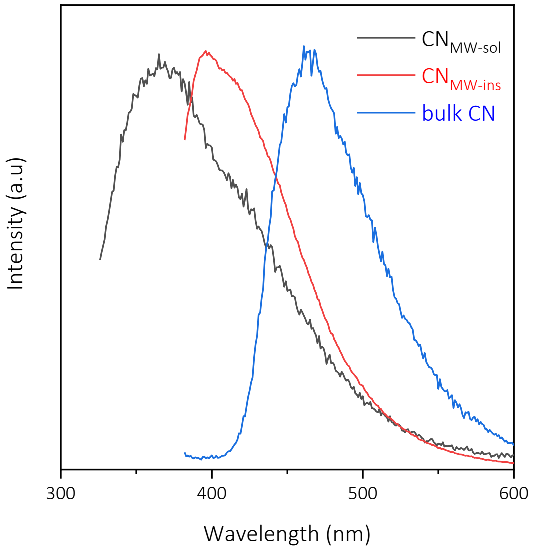

Supplementary Figure 12 Normalized photoluminescence spectra of CN_{MW-sol} (E_x = 311 nm), CN_{MW-ins} and bulk CN (E_x = 367 nm) powders.

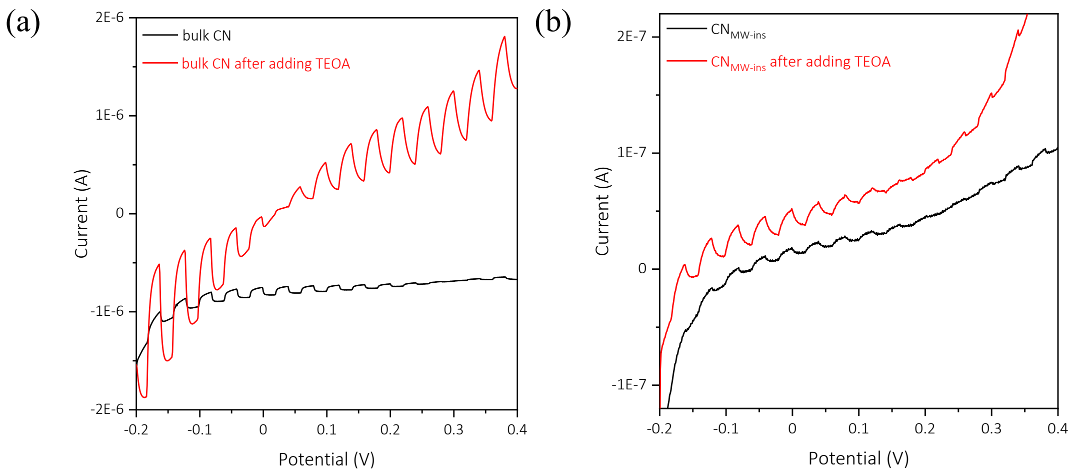

Supplementary Figure 13 Current–potential curve of bulk CN and $CN_{MW\text{-}ins}$ before and after adding the electron donor (triethanolamine, TEOA, 10 v/v%) in 0.1 mM KCl under chopped light.

As $CN_{MW\text{-}ins}$ contained more M2 than bulk CN, it indicated that M2 played an important role in accelerating the charge separation. The hole- and electron-extraction properties were also evaluated by measuring anodic and cathodic photocurrents, respectively, in the presence of an electron donor (triethanolamine, TEOA), assuming the maximum photocurrent can be obtained without any hole- or electron-transfer limitations.[11, 12] As shown in Supplementary Figure 13, both anodic and cathodic photocurrent at $CN_{MW\text{-}ins}$ were much less improved with respect to bulk CN by adding TEOA, indicating a superior hole- and electron-extraction properties contributed by M2.

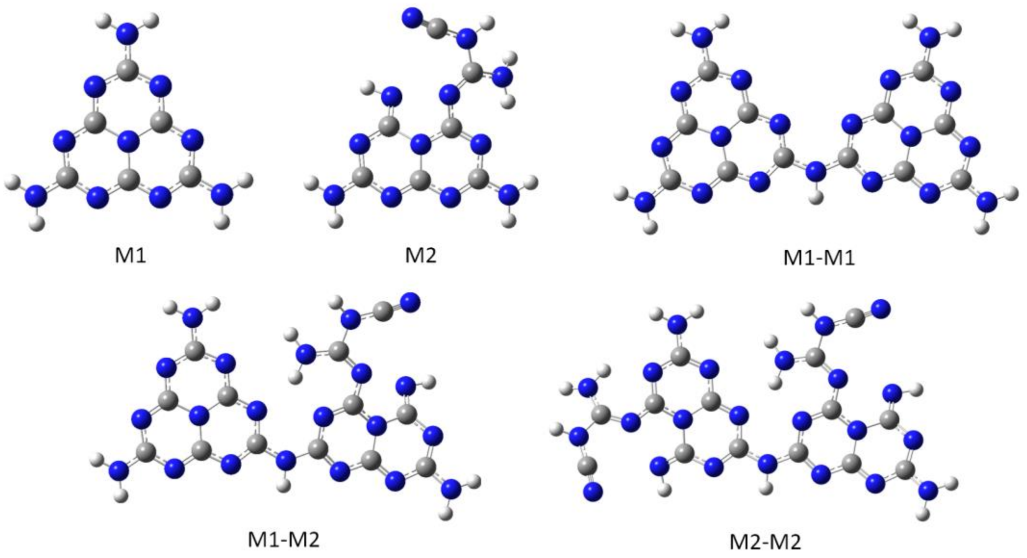

Supplementary Figure 14 Optimized geometries of CN molecules at M06-2X/6-31G(d,p) level.

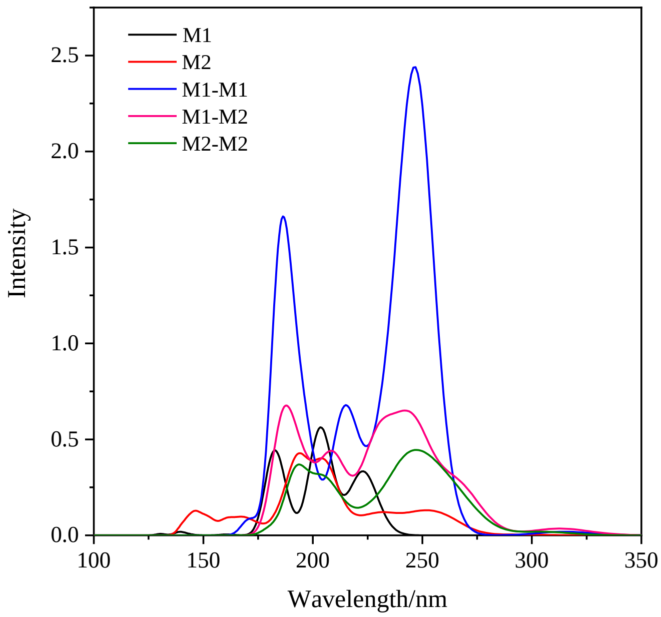

Supplementary Figure 15 Absorption spectra of all CN molecules calculated at M06-2X/6-31G(d,p) level.

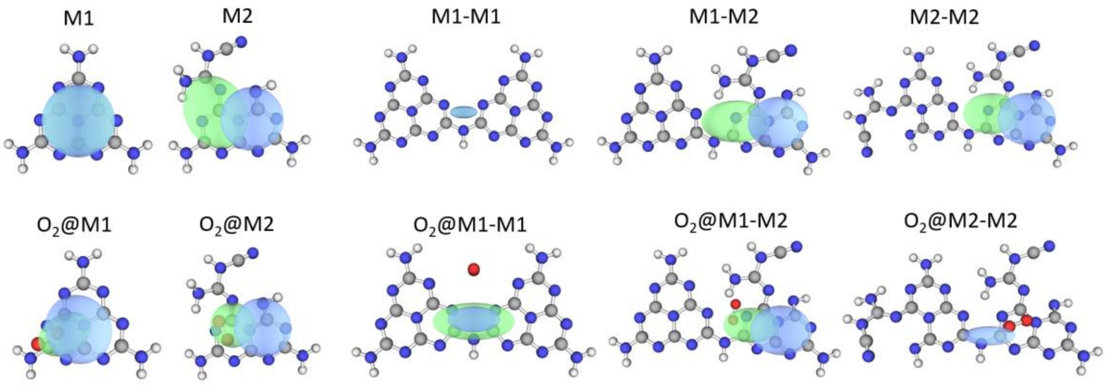

Supplementary Figure 16 Smooth description of spatial distribution of electron and hole population in S_1 of different CN molecules and O_2@CN systems. The green and blue color represent for the electron and hole, respectively.

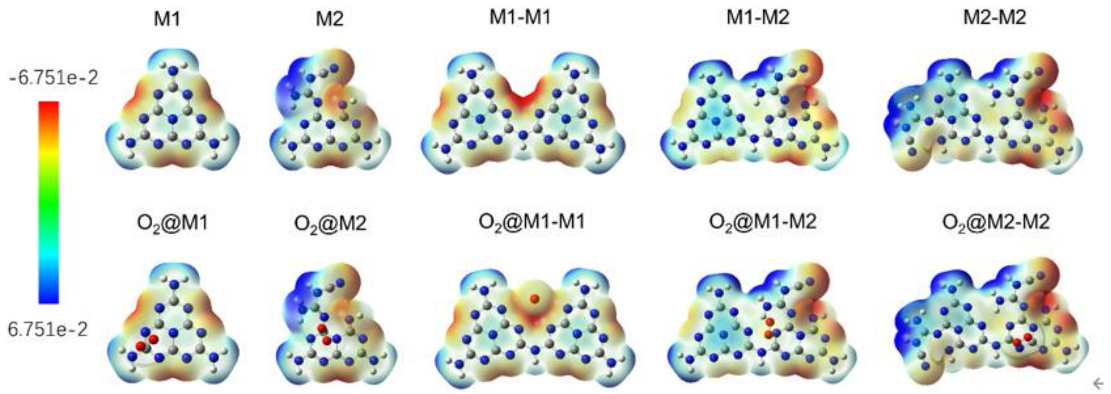

Supplementary Figure 17 Electrostatic surface potentials (unit: a. u.) mapped onto a surface of total electron density (the isosurface threshold of electron density: 0.001) for all the investigated molecules.

The electrostatic surface potential (ESP) of the ground states of the investigated molecules are shown in Supplementary Figure 17. The red, green and blue colors represent negative charge, neutral region and positive charge, respectively. For all systems, the positive charge is mainly distributed on the hydrogen atoms, and the negative charge is located on the nitrogen atoms of the conjugated ring, which can be attributed to the strong electron-withdrawing feature of the nitrogen atom. Due to the high symmetry of M1, the centers of positive and negative charges almost completely overlap. In contrast, the distance between the positive and negative charge centers of M2 become larger owing to the asymmetric structure, which is conducive to the spatial separation of holes and electrons. In addition, the positive and negative charges in M2 become larger as the color of the positive and negative charge regions deepens. The symmetrical M1-M1 and asymmetrical M1-M2/M2-M2 also show similar charge distribution, indicating that M2 with an asymmetric structure is more likely to be oxidized/reduced than M1, and thus charge transfer occurs easily with O_2. Consequently, M2 may be more conducive to the activation of O_2, which is consistent with our TDDFT analysis of the electron-hole separation in the first excited state (see Supplementary Figure 16).

Supplementary Table 1 Elemental analysis of bulk CN, Melamine, $CN_{MW\text{-}ins}$ and $CN_{MW\text{-}sol}$

Sample	C [wt. %]	N [wt. %]	H [wt. %]	C/N [molar ratio]	theor. C/N [molar ratio]
Bulk CN	34.66	59.22	1.955	0.68	0.75
$CN_{MW\text{-}ins}$	30.94	56.06	4.098	0.64	-
$CN_{MW\text{-}sol}$	29.28	58.92	4.990	0.58	-
Melamine	28.65	65.03	4.861	0.51	0.50

Supplementary Table 2 Centroids distance of electron-hole (D), overlap integral (S_r), index of separation degree (t) and coulomb attraction energy (E_c) of S_1 in different CN and O_2@CN systems.

	D (Å)	S_r	t (Å)	E_c (eV)
M1	0.000	0.504	-1.765	5.846
M2	2.350	0.489	0.821	5.062
M1-M1	0.023	0.548	-2.187	4.066
M1-M2	2.976	0.493	0.835	4.360
M2-M2	2.698	0.503	0.615	4.550
O_2@M1	1.244	0.502	-0.205	5.311
O_2@M2	1.963	0.528	0.543	5.098
O_2@M1-M1	0.140	0.633	-1.473	4.424
O_2@M1-M2	2.087	0.622	-0.067	4.813
O_2@M2-M2	1.054	0.689	-1.799	4.171

Supplementary References

1. Wang, J., Shen, Y., Li, Y., Liu, S. & Zhang, Y. Crystallinity modulation of layered carbon nitride for enhanced photocatalytic activities. *Chem. Eur. J.* **22**, 12449-12454 (2016).
2. Wilson, P., Bradley, T. & Tozer, D. Hybrid exchange-correlation functional determined from thermochemical data and ab initio potentials. *J. Chem. Phys.* **115**, 9233-9242 (2001).
3. Zhou, Z. et al. Dissolution and liquid crystals phase of 2D polymeric carbon nitride. *J. Am. Chem. Soc.* **137**, 2179-2182 (2015).
4. Yu, H. et al. Alkali-assisted synthesis of nitrogen deficient graphitic carbon nitride with tunable band structures for efficient visible-light-driven hydrogen evolution. *Adv. Mater.* **29**, 1605148 (2017).

5 Zhang, Y., Thomas, A., Antonietti, M., & Wang, X. Activation of carbon nitride solids by protonation: morphology changes, enhanced ionic conductivity, and photoconduction experiments. *J. Am. Chem. Soc.* **131**, 50-51 (2009).

6 Zhang, X. et al. Enhanced photoresponsive ultrathin graphitic-phase C_3N_4 nanosheets for bioimaging. *J. Am. Chem. Soc.* **135**, 18-25 (2013).

7. Huang, C. et al. Dissolution and homogeneous photocatalysis of polymeric carbon nitride. *Chem. Sci.* **9**, 7912-7915 (2018).
8. Han, Q., Wang, B., Zhao, Y., Hu, C.G. & Qu, L.T. A graphitic C_3N_4 seaweed architecture for enhanced hydrogen evolution. *Angew. Chem. Int. Ed.* **54**, 11433-11437 (2016).
9. Zhen, W. et al. Efficient visible-light-driven selective oxygen reduction to hydrogen peroxide by oxygen-enriched graphitic carbon nitride polymers. *Energy Environ. Sci.* **11**, 2581-2589 (2018).
10. Cai, R. et al. Single nanoparticle to 3D supercage: framing for an artificial enzyme system. *J. Am. Chem. Soc.* **137**, 13957-13963 (2015).
11. Zhao, T. et al. Ultrafast condensation of carbon nitride onelectrodes with exceptional boosted both photocurrent and electrochemiluminescence. *Angew. Chem. Int. Ed.* **59**, 1139-1143 (2020).
12. Peng, G., Albero, J., Garcia, H. & Shalom, M. Water-splitting carbon nitride photoelectrochemical cell with efficient charge separation and remarkably low onset potential. *Angew. Chem. Int. Ed.* **57**, 15807-15811 (2018).
